# Serological findings following the second and third SARS‐CoV‐2 vaccines in lung transplant recipients

**DOI:** 10.1002/iid3.646

**Published:** 2022-07-25

**Authors:** Enikő Bárczi, Viktória Varga, Alexandra Nagy, Noémi Eszes, Zsuzsanna Jáky‐Kováts, Veronika Müller, Anikó Bohács

**Affiliations:** ^1^ Department of Pulmonology, Faculty of Medicine Semmelweis University Budapest Hungary

**Keywords:** COVID‐19, mRNA vaccine, non‐mRNA vaccine, pandemic, SARS‐CoV‐2, third vaccination, transplant, vaccine

## Abstract

**Introduction:**

Lung transplant recipients (LuTX) represent a vulnerable population for severe acute respiratory syndrome coronavirus 2 (SARS‐CoV‐2). Even though many vaccines are already developed, more clinical data need to support effective immunological response in immunocompromised patients.

**Methods:**

Stable LuTX recipients with no medical history of coronavirus disease (COVID‐19) were enrolled. Currently available messenger RNA (mRNA) (BNT162b2‐mRNA, mRNA‐1273) and non‐mRNA (ChAdOx1, BBIBP‐CorV) vaccines were given according to availability, boosters were all mRNA‐based. SARS‐CoV‐2 Spike1 immunoglobulin G (IgG) antibody titer was evaluated before and 2 weeks after second and third dose. Difference between mRNA versus non‐mRNA vaccines was assessed.

**RESULTS:**

Forty‐one patients (49% men, age 48.4 ± 13.8 years) received two doses of SARS‐CoV‐2 vaccines: 23 of mRNA, 18 of non‐mRNA, and 24/41 (58%) received a third dose. Median 92 months passed since transplantation, and serum level of tacrolimus was median 5.5 ng/ml. Positive serology was found in 37% of all patients after the second dose, 86% had mRNA vaccine. After the third dose, 29% became positive who had no antibody before. Significantly higher level of antibody was found after the second mRNA than non‐mRNA vaccines (2.2 vs. 1568.8 U/ml, respectively, *p* = .002). 6/23 (26%) patients received two doses of mRNA vaccine developed COVID‐19 after the second injection in an average of 178 days, half of them recovered, half of them died in intensive care unit (ICU). 3/6 (50%) patients with two doses mRNA and recovered from COVID‐19 had significantly higher level of antibody (average 20847.3 U/ml) than without infection. After the booster vaccine, 1/24 (4%) developed infection.

**Conclusion:**

Immunosuppression therapy may induce a weaker SARS‐CoV‐2 response in LuTX recipients; therefore, third dose is a priority in transplanted patients. The highest antibody level was measured recovering from COVID after two doses. Our data confirm that booster mRNA vaccine could increase antibody levels, even if immunization was started with non‐mRNA vaccine.

## Graphical abstract

Serological findings following SARS CoV 2 vaccines in lung transplant recipients

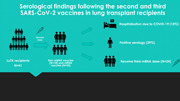

## 1 INTRODUCTION

Solid organ recipients, especially lung transplant (LuTX) patients are of high risk for airborne viral infections, including the new coronavirus severe acute respiratory syndrome coronavirus 2 (SARS‐CoV‐2). As LuTX patients receive high doses of immunosuppressant medications, they are at very highest risk of developing severe coronavirus disease 2019 (COVID‐19) and increased mortality.^1^ Since the beginning of the pandemic vaccination is the key for population scale prevention of severe infection. The major target of vaccines is the viral spike protein which through its receptor‐binding domain interacts with the human angiotensin‐converting enzyme 2 receptor (ACE‐R2) and the interaction enables viral entry into human epithelial cells. Currently approved vaccines stimulate both B‐ and T‐cell responses, engaging both humoral and cellular immune pathways.^2^ Previous studies have shown that transplanted patients infected with SARS‐CoV‐2 have a worse outcome than the general population,^3^, ^4^ therefore prevention plays the most important role in the protection of LuTX recipients. We have a lack of knowledge which type of vaccine is the best for immunocompromised patients. Most of the studies used mRNA‐based vaccines, and they mostly reported severely impaired antibody response to mRNA vaccines.^5^, ^6^, ^7^

Transplant recipients were excluded from recent SARS‐CoV‐2 vaccine trials, so there are insufficient data about efficacy, durability, or safety in this patient subpopulation. Our study aims to assess specific immunological response to different types of vaccinations and determine the proportion of seroconverted patients and outcomes in Hungarian LuTX patients.

## 2 METHODS

### 2.1 Transplantation protocol

Before 2015 our patients underwent lung transplantation in Vienna. The first lung transplantation procedure was performed in Budapest, Hungary on December 12,  2015. The Department of Pulmonology, Semmelweis University is the only center for LuTx posttransplant care in Hungary. Our transplant activity is 20 cases per year. Our center uses the immunosuppression protocol of Medical University of Vienna. The induction therapy was antithymocyte globulin (ATG) of patients who underwent lung transplantation before 2008. From 2009 monoclonal antibody targeting CD52 (alemtuzumab) 30 mg intravenously was the induction therapy followed by lower maintenance immunosuppression.^8^ Calcineurin inhibitor (tacrolimus) blood level was 8–10 ng/ml after LuTx 0–3 months, 6–8 ng/ml at 3–12 posttransplant month (PTM), 5–7 ng/ml at 12–24 PTM, and 5 ng/ml at > 24 PTM. Systemic steroids were tapered in the first year 0.2 mg/kg prednisolone at 0–3 PTM, 0.15 mg/kg at 3–6 PTM, 0.1 mg/kg at 6–12 PTM, 5 mg/day after 1 year. Mycophenolate‐mofetil (1–1.5 g twice daily) was initiated 12 months after LuTx.^9^ LuTx patient who has got bronchiolitis obliterans syndrome or malignancy the immunosuppressive treatment was changed, along the tacrolimus mTOR inhibitor (everolimus) was started. Mycofenolate‐mofetil was stopped in patients with posttransplant malignancy.

### 2.2 Study protocol

LuTX patients in the study period between January and December 2021 included 148 individuals. Patients were recruited at least 1 year after the surgery, if they were in stable functional condition, and if no rejection nor infection was present. Those who had immunoglobulin deficiency were excluded. In Hungary, according to actual regulations individuals who were infected with SARS‐CoV‐2 within the last 3–6 months were exempt from vaccinations, including 30 LuTX patients.

Out of the potentially eligible 118 patients, 41 volunteered for baseline SARS‐CoV‐2 IgG and IgM measurements at study entry to help determine baseline SARS‐CoV‐2 serostatus for the analysis of vaccine efficacy and were approved by local ethical committee (TUKEB‐IV/861‐1/2021/EKU).

At the time of the study BNT162b2, mRNA‐1273, ChAdOx1, BBIBP‐CorV, and Gam‐COVID‐Vac vaccines were approved in Hungary. As there was no recommendation which vaccine should LuTX patients receive, all were vaccinated by the offered and available vaccines at centrally assigned vaccination points. SARS‐CoV‐2 Spike1 IgG antibody (Roche®) titer was measured 2 weeks after the second dose of the respective vaccine and if available, after the third dose at their regular control (2–6 weeks postvaccination). Positive serology was considered >0.8 U/ml serum level of anti‐SARS‐CoV‐2‐Spike1.

### 2.3 Statistical analysis

Statistical analysis was performed using Graph Pad software (GraphPad Prism 5.0 Software, Inc.). Data are expressed as mean ± standard deviation (SD) or median. Differences between groups for parametric data were evaluated with Student's *t* test after testing for normality using Kolmogorov–Smirnov test, while *χ* ^2^ test was applied for analyzing categorical data. A *p* value <.05 was defined as statistically significant.

## 3 RESULTS

Forty‐one LuTX recipients were enrolled into the analysis. Baseline characteristics are summarized in Table 1. Median age was 48.4 ± 13.8 years (interquartile range [IQR]: 19– 70 years), more women than men register voluntarily for vaccination. Most of the patients were transplanted due to cystic fibrosis (42%) and median 92 months (IQR: 10–256 months) have passed since transplantation. During the initial vaccination 56% received mRNA‐based vaccines, most of them had two doses of BNT162b2 (*N* = 20). The most common non‐mRNA vaccine was ChAdOx1 (N = 16). Twenty‐four of 41 (58%) patients got third dose after the second one on average 177 ± 42 days later, all third doses were mRNA‐based. No serious adverse events other than general lethargy and local skin reaction were recorded neither after the second nor the third vaccination.

**Table 1:** Characteristics of lung transplant recipients according to the type of vaccination at primary

Parameter	All patients (*N* = 41)	mRNA vaccine (*N* = 23)	non‐mRNA vaccine (*N* = 18)	*p*
Age: years	48.4 ± 13.8	50.1 ± 15.6	46.26 ± 11.3	ns
Gender: N				
Male:female	20:21	14:9	6:12	ns
Indication of transplantation: N (%)				
COPD (chronic obstructive pulmonary disease)	11 (27)	7 (30)	4 (22)	ns
IIP (idiopathic interstitial pneumonia, e.g. idiopathic pulmonary fibrosis)	10 (24)	6 (26)	4 (22)	ns
CF (cystic fibrosis)	17 (42)	8 (35)	9 (50)	ns
PAH (pulmonary arterial hypertension)	3 (7)	2 (9)	1 (6)	ns
Median time from transplantation: months [range]	92 [10–256]	78.8 [15–175]	106.5 [10–256]	ns
COVID‐19 infection after second vaccine: *N* (%)	6 (15)	6 (23)	0	0.06
Average days [range]	‐	178 [163‐206]	‐	‐
Type of vaccine: *N* (%)				
BNT162b2 (Pfizer‐BioNTech)	‐	20 (87)	‐	‐
mRNA‐1273 (Moderna)	‐	3 (13)	‐	‐
ChAdOx1 (Astra)	‐	‐	16 (89)	‐
BBIBP‐CorV (Sinopharm)	‐	‐	2 (11)	‐
Patients with positive serology after the second dose: *N* (%)		13 (57)	2 (11)	0.002

Abbreviation: COVID‐19, coronavirus disease 2019.

Eighteen (*n* = 18) recipients received ATG as induction therapy and 23 alemtuzumab. Each patient received tacrolimus and prednisolon. The median dosage of prednisolone was 5 mg/day. The median tacrolimus serum level was 5.5 ng/ml (IQR: 2.5–11.2 ng/ml) before the first vaccination.

Eleven out of 41 patients (26%) were treated with tacrolimus+everolimus+prednisolone. The total serum level of tacrolimus and everolimus was median 6.6 ng/ml (IQR: 3.6–11.2 ng/ml). Each patient except seven cases received mycophenolate‐mofetil, median 1500 g/day (IQR: 500–2000 g/day). Under the vaccination period, the immunosuppressive treatment did not change.

Immune response for SARS‐CoV‐2‐Spike1 was not measurable in most cases after the second dose (serum level was <0.8 ng/ml, *N* = 26; 64%), 3/41 patients (7%) had low‐positive antibody level (<10 U/ml) and six patients (15%) developed >1000 U/ml antibody titer 2 weeks after second vaccine. Thirteen out of 23 (57%) mRNA‐vaccinated patients became seropositive after two shots. Eighteen out of 41 patients received two doses of non‐mRNA vaccine; positive serology was found only in two cases (11%). A significant difference was found between the response of mRNA versus non‐mRNA vaccines (average 1568.8 U/ml vs. 2.2 respectively, *p* = .002), and the highest immune responses (anti‐Spike1 level: 2709, 1918, 1170 U/ml) were found in patients vaccinated with two doses of BNT162b2.

Thirteen recipients out of the 24 who received three doses (54%) still did not develop any immune response neither after the second nor the third vaccination. However, seven patients (29%) had positive antibody after the third dose who had none before and in these patients the average antibody titer was 2435 U/ml. Five of them received ChAdOx1, two of them BNT162b2 vaccines.

**Figure 1:**
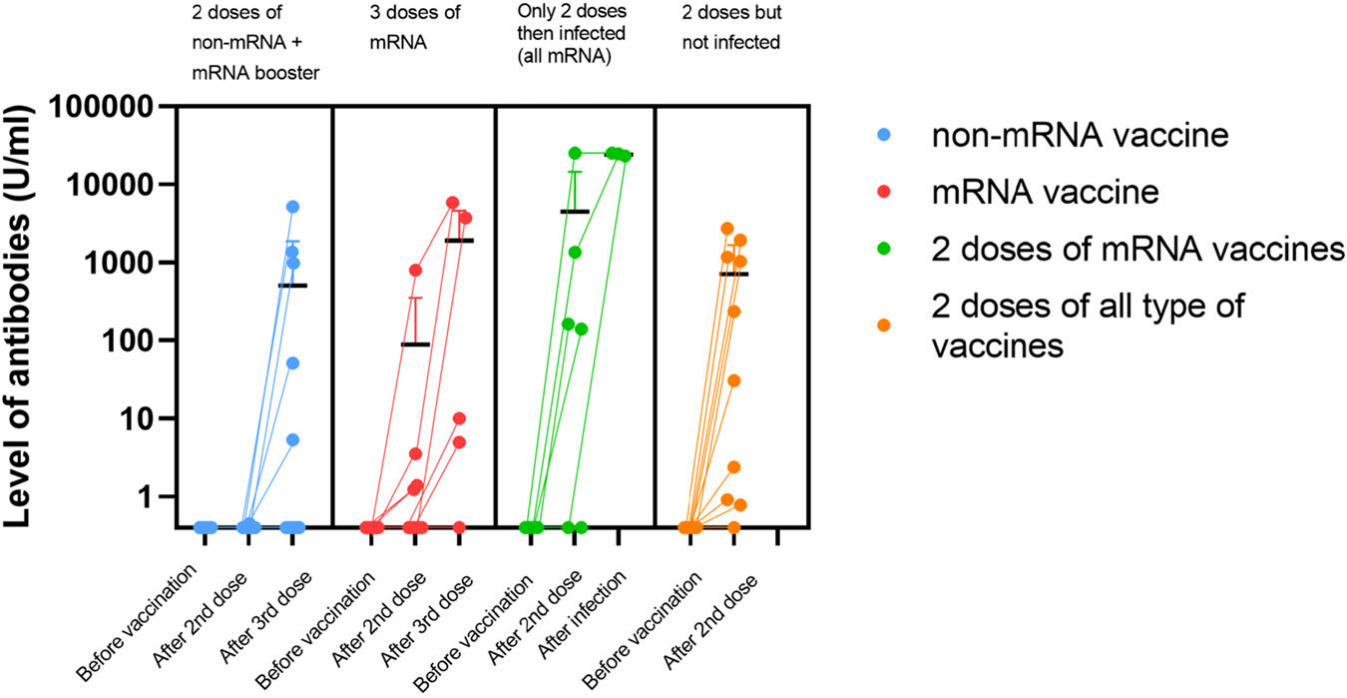
Level of SARS‐CoV‐2 Spike1 antibodies (U/ml) differentiated by vaccination types. The third column shows antibody levels in patients infected with SARS‐CoV‐2 after vaccinated two times. There was a significant difference between mRNA versus non‐mRNA vaccine‐induced immune response (*p* = .002), and antibody response is significantly higher (*p* < .05) in recovered patients after two doses of injections. All infected patients received mRNA vaccine. Three patients died due to COVID, their third antibody level is missing. As the primary immunization mRNA vaccines were BNT162b2‐mRNA and mRNA‐1273, while non‐mRNA vaccines were ChAdOx1 and BBIBP‐CorV. The booster was mRNA vaccine in every case. COVID, coronavirus disease; mRNA, messenger RNA; SARS‐CoV‐2, severe acute respiratory syndrome coronavirus 2.

Six patients developed SARS‐CoV‐2 infection after the second vaccination in an average of 178 days, all of them received BNT162b2. Three patients had no detectable antibody, while the other three had 140, 160, and 1346 U/ml antibody levels respectively after two doses of vaccination. Significantly higher antibody levels were detected after recovering from infection (13052, 24990, >25000 U/ml) than after two doses of vaccines (average level: 244 U/ml [0.4–2709 U/ml]; *p* = .05). Only one of them was asymptomatic and recovered at home, while the other five required hospitalization. Two patients had moderate disease with 10%–20% involvement of the lungs, after a short time of hospitalization they recovered with no functional loss and high antibody titer (>10,000 U/ml) was measured thereafter. Three out of six patients had severe illness and needed intensive care, where they died soon, and after their second vaccination 183, 186, and 216 days have passed, respectively. Figure 1 shows antibody levels according to the vaccination and infection status. One patient had mild COVID‐19 after the booster vaccine, however, he had no detectable antibody level after any vaccination.

## 4 DISCUSSION

Transplant recipients may be at high risk for COVID‐19 due to chronic immunosuppressive treatment and other medical comorbidities.

Our data confirmed low seroconversion rate following two doses of non‐mRNA SARS‐CoV‐2 vaccine (11%) while 57% of the patients became seropositive after two doses of mRNA vaccines. Among all patients vaccinated three times, positive antibody response increased from 17% to 46%, with a corresponding increase in neutralizing capacity after the third dose. The highest individual antibody level (>25000 U/ml) was measured after two doses of mRNA vaccines, and then recovering from SARS‐CoV‐2 infection. Infection could be life‐threatening in transplant recipients. In our patient population mortality was high (50%) among infected recipients, even though two or three doses of vaccines. No serious side effects of vaccination were observed. In the most recent recommendation, third dose is theoretically suggested, but that was not yet available in many countries.^10^, ^11^ A previous study with different type of vaccines had shown, that 67% of the solid organ transplanted (SOT) patients (especially liver and kidney recipients) remains negative after a booster vaccine which was given in a median of 67 days.^12^ Most studies dealing with mRNA‐based vaccinations in SOT recipient, and contribute to a small number of lung transplant patients.^7^, ^13^ They all suggested that immune response remains low after two doses of mRNA vaccine but neutralizing antibody levels improved after the booster vaccine.^12^, ^14^, ^15^, ^16^

Several studies exists who studied the effectiveness of the booster vaccines in heart‐ transplanted patients,^17^ kidney transplant recipients,^18^ or other solid organ recipients^12^ but only a few and with low number of cases exist in lung transplant patients.^19^ A previous study showed that none of the LuTX recipients had immune response to two doses of BNT162b2 (*N* = 48); however, 85% of the patients had high antibody response after COVID‐19.^5^

The low antibody response rate is alarming but not unexpected in LuTX recipients. Previous experiences with Influenza vaccine show a lower rate of immune response in solid organ recipients, however, vaccination demonstrated reduced influenza‐related lower respiratory tract disease and hospitalization despite low antibody response.^20^ Lung transplant recipients are at the greatest risk of rejection among all solid organ recipients, therefore immunosuppressive agents should be changed carefully. Immunosuppressants may partly cause the weak immune response because of altered T lymphocyte functions, however, dose reduction increases the risk of graft rejection.^21^ Recommendations suggest not to reduce and hold immunosuppressant before SARS‐CoV‐2 vaccination.^22^

Other concerns regarding the SARS‐CoV‐2 vaccines include the lack of long‐term safety data, potential reduction in efficacy in immunocompromised patients, unknown durability of the immune response, and potential for vaccine‐associated allograft rejection. Despite the low proportion of seroconversion observed in our patients, SARS‐CoV‐2 vaccines provide potential benefit and decreases the risk stabling transplant recipients.

We know our study limitations with the low number of the cases, and vaccine efficacy cannot be defined obviously by the presence or absence of antibodies, which may be deceptive. We should also note that in lines with positive antibody response there is still no universal cutoff value. However, we observed low antibody response in patients vaccinated with non‐mRNA vaccines, none of them get COVID‐19. Additionally to neutralizing antibodies T helper1 CD4 + and CD8 + T cell response might contribute to immunity against SARS‐CoV‐2. Further data are needed to evaluate B‐ and T‐cellular responses after SARS‐CoV‐2 vaccination and disease.

The emerging studies on SARS‐CoV‐2 vaccines indicate that they are safe, and our experience with different SARS‐CoV‐2 vaccines proved to be safe in lung transplant recipients. Currently, the third booster dose of SARS‐CoV‐2 vaccine is the most appropriate way to increase the immune response among lung transplant recipients. The durability of this immune response should be evaluated in the need of additional boosters in the future. However, we still have insufficient amounts of data using non‐mRNA‐based vaccines among transplant recipients, there is promising result about switching to a different type of vaccine (e.g., viral vector‐based vaccination after mRNA) in nonresponders thereby offering a new strategy to increase immune response. Vaccination is the most promising way to tackle the pandemic, and third or more doses of vaccine are becoming a chance to avoid serious COVID‐19 complications.

## AUTHOR CONTRIBUTIONS

All authors have read and approved the final manuscript. All authors performed the research. Enikő Bárczi, Anikó Bohács, and Veronika Müller designed the research study, analyzed the data, and wrote the paper.

## CONFLICTS OF INTEREST

The authors declare no conflicts of interest.

## Data Availability

Data that support the findings of this study are available from the corresponding author upon reasonable request.
